# Effect of Lactic Fermentation and Cooking on Nutrient and Mineral Digestibility of Peas

**DOI:** 10.3389/fnut.2022.838963

**Published:** 2022-02-24

**Authors:** Sylvie Skalickova, Andrea Ridoskova, Petr Slama, Jiri Skladanka, Petr Skarpa, Iva Smykalova, Jiri Horacek, Radmila Dostalova, Pavel Horky

**Affiliations:** ^1^Department of Animal Nutrition and Forage Production, Faculty of AgriSciences, Mendel University in Brno, Brno, Czechia; ^2^Department of Chemistry and Biochemistry, Faculty of AgriSciences, Mendel University in Brno, Brno, Czechia; ^3^Department of Animal Morphology, Physiology and Genetics, Faculty of AgriSciences, Mendel University in Brno, Brno, Czechia; ^4^Department of Agrochemistry, Soil Science, Microbiology and Plant Nutrition, Faculty of AgriSciences, Mendel University in Brno, Brno, Czechia; ^5^Agritec Plant Research, Ltd., Šumperk, Czechia

**Keywords:** iron, proteins, amino acids, polyphenol compounds, biotechnology, phytic acid, raffinose, starch

## Abstract

Peas are prospectively beneficial legumes in the human diet, and especially in a vegan and vegetarian diet, due to their high content of proteins and starch. Their frequent lack of appeal in human nutrition can be caused by their bloating effect and the content of some antinutritional compounds inhibiting the absorption of important nutrients. This study brings a comprehensive comparison of the nutrient content of pea flour after cooking and lactic fermentation before and after digestion *in vitro*. As a control sample, raw pea flour was used (sample 1). Raw pea flour was cooked for 10 min (sample 2) and 120 min (sample 3) at 100°C or it was fermented by *Lactobacillus plantarum* (sample 4) and cooked for 10 min at 100°C (sample 5). The samples were analyzed for protein and amino acids content, maltose, glucose, raffinose, total polyphenols, phytic acid, phytase, and mineral composition (P, Mg, Mn, Fe, Cu, Zn) before and after *in vitro* digestion. The results showed a significant (*p* < 0.05) increase in the protein digestibility of samples 3, 4 and 5. In the fermented samples were observed a higher concentration of Cys, Met, and Gln when compared to non-fermented samples. The fermentation of pea flour resulted in a significant (*p* < 0.05) decrease in glucose, maltose, and raffinose content. Cooking of pea flour for 10 and 120 min, but not fermenting, significantly (*p* < 0.05) decreased the polyphenols content. Cooking and fermentation together did not affect phytic acid concentration and phytase activity. Mg, Mn, Fe, Cu and, Zn concentration in pea flour was significantly (*p* < 0.05) decreased by cooking. On the other hand, fermentation significantly (*p*<0.05) improved the bioaccessibility of Mn and Fe. These findings suggest that lactic fermentation of pea flour is a promising culinary preparation that can improve the digestibility of peas.

## Introduction

Globally, there is a growing demand for non-animal protein stemming from the ethical or ecological concerns that meat production produces a higher carbon footprint compared to plant production (1). Plant proteins are a promising and widely used substitution for animal protein, especially in vegan and vegetarian diets. Plant-based foods are distinguished by high content of fiber, mineral compounds, and vitamins; moreover, they have a low content of fat and are cholesterol-free. These nutritional advantages make plant proteins beneficial in terms of health and prevention of lifestyle diseases (2). However, there are disadvantages from the point of view of human nutrition: a low proportional representation of essential amino acids, low bioavailability of iron, cobalamin deficiency, presence of dietary estrogen, and a high content of antinutritional compounds, e.g., phytic acid, tannins, or trypsin inhibitors, which debase the bioavailability of essential nutrients (3). All of these shortcomings can be addressed with animal-based food which operate in symbiotic ways with plant-based food, but at the cost of a larger carbon footprint (4). This fact motivates scientists and biotechnology companies to pursue further research and development of plant-proteins production toward the goal of increasing the bioavailability of essential nutrients and improving plant-protein nutritional quality for human nutrition.

Bean, soybean, and chickpea production globally dominate in the world's market of pulses. The fourth most popular crop is the pea. North America, Europe, and east Asia are the biggest producers, although its production has seen a significant decrease in recent years due to increased soybean production (5, 6). However, the pea excels in nutritional composition, higher content of lysine and tryptophane, and lower content of trypsin inhibitors compared to the soybean. Nevertheless, the pea is more commonly used in animal nutrition than for humans (7). In terms of human nutrition, the consumption of peas is strongly influenced by a consumer choice. With widespread trends such as low-carb diet and vegan/vegetarian movement, the value of the pea as a source of protein is growing within consumers as well as the food industry (8). For example, in recent years, extracted pea protein, which can be directly consumed or is a part of food such as bread or soups, has been introduced to the global market. Its benefit is the absence of fermentable oligosaccharides and antinutritional compounds, as well as better digestibility (9).

In human nutrition, peas are mostly consumed as a fresh vegetable or cooked dish in the form of porridge, soup, or bread. Some consumers are deterred from peas not only for their sensory properties (taste and smell typical of peas) and the content of antinutritional compounds (phytic acid, tannins, and trypsin inhibitors), but also the content of fermentable oligosaccharides which cause bloating: mainly raffinose, stachyose, verbascose, and sucrose (10). A small fraction of these oligosaccharides can be eliminated by the treatment of pea flour at high temperatures, extrusion, or soaking of dried peas before processing; however, residual oligosaccharides are still present in peas and can cause digestive problems (11). In addition, the presence of antinutritional compounds makes peas less digestible. Phytic acid naturally occurs in legumes and causes a decreased bioavailability of essential minerals such as zinc, phosphorus, iron, and others. Trypsin inhibitors and tannins can also result in lower digestibility of proteins (11).

The technology available today possesses a prospective way to eliminate antinutritional compounds, increase the protein digestibility, and improve the bioavailability of essential amino acids in plant-based foods (12). The most promising approach is fermentation of the plant-based food by lactic bacteria (13). The genus Lactobacillus is found in different environments including milk, the gastrointestinal tract (GIT), fermented meat, dairy products, and cereal-based foods (14). Currently, extensive research is being performed on a member of Lactobacillus genus, *L. plantarum*, in terms of food fermentation. This has shown that *L. plantarum* utilizes fermentable oligosaccharides and decomposes starch as well as retrograded starch. Moreover, its positive effects on fermented food has been shown, such as an antimicrobial effect (15), a protective effect on food against pathogenic molds (16), production of lactic acid and B-vitamins, as well as acting as a probiotic strain in the GIT. With regards to usage of *L. plantarum* as a probiotic supplementation, Kyereh et al. have observed that the mixture of starchy food, such as maize, soybean, and cowpea, prolonged the survival of *L. plantarum* in the GIT (17). In another study, *L. plantarum* has shown the ability to eliminate unpleasant aroma in pea protein isolates (18). Taken together, the biotechnological research and development of pea flour fermentation is a promising tool for improving the nutritional value of peas and underscoring the benefits of peas in human nutrition.

The aim of this study was to provide a comprehensive view of the digestibility of proteins and starch and determine the content of antinutritional compounds (raffinose, phytic acid, polyphenols), as well as mineral elements in cooked and fermented pea flour by *Lactobacillus plantarum*. This study compares 10 min cooking time as a simulation of preparation of instant pea flour product, and 120 min cooking time as a juxtaposing extreme-long time to compare changes on its digestibility.

## Materials and Methods

### Chemicals and Reagents

Inorganic salts used in the *in vitro* digestion assay and other chemicals, unless noted otherwise, were purchased from Sigma Aldrich (USA). Rabbit gastric lipase was purchased from Lipolytech (Marseille, France). *L. plantarum* (ATCC 8014) was obtained from the Czech Collection of Microorganisms, Faculty of Science, Masaryk University, Brno, Czech Republic. The pH value was measured using pH 60 VioLab, (XS instruments, Via della Meccanica, Italy). Deionized water underwent demineralization by reverse osmosis using the instruments Aqua Osmotic 02 (Aqua Osmotic, Tisnov, Czech Republic).

### Pea Flour Samples

Pea flour samples were provided by Pro-Bio s.r.o. (Czech Republic). Peas, ESO variety, were grown in western Bohemia, Domazlice district, the Czech Republic in the altitude around 400 MSL. The average annual temperature during germination was 7–8°C and average total precipitation was 600 mm. The peas were grown with barley and fertilized with manure. Before sowing, PKS fertilizer was applied at a rate of 200 kg/ha. The peas were harvested in August 2020, separated from the barley and then dried. The peas were dehulled and ground to particle size 256 microns. The nutritional composition of raw pea flour is shown in Table 1. Nutritional analyses were carried out according to ISO/IEC 17025:2017.

**Table 1:** Nutritional composition of raw pea flour.

**Nutrient**	**% of dry matter**
Dry matter	89.89
Ash	2.59
Nitrogen compounds	19.95
Crude fat	1.22
Crude fiber	0.45
ADF	2.23
NDF	10
ADL	0
Celulose	2.23
Starch	59.03

### Sample Preparation

*L. plantarum* was anaerobically cultivated on MRS agar (Hi-Media, Mumbai, India) at 37°C, 120 rpm for 72 h on thermostated shaking incubator (BioSan, Latvia). Ten grams of pea flour in 89.5 mL of deionized, sterile water were inoculated with 500 μL of diluted bacterial suspension in deionized sterile water (1.5 x 10^8^ CFU/mL). Fermentation of pea flour was carried out for 72 h at 37°C aerobically on thermostated shaking incubator (Biosan, Latvia).

Ten grams of pea flour were dissolved in 90 mL deionized sterile water for all treatments. Sample without any treatment was used as a control (1). For the first (sample 2) and the second treatment (sample 3), the samples were cooked at 100°C for 10 or 120 min under continuous stirring. The fourth treatment was fermented pea flour without thermal treatment (sample 4) and the fifth sample was fermented pea flour (5) that was cooked at 100°C for 120 min. The thermal treatment was carried out on the magnetic stirrer under continuous stirring (650 rpm) with an external temperature probe (Intelli-Stirrer MSH-300i, Biosan, Latvia). Samples were cooled down and subsequently sterile and deionized water was added for weight balancing of all the samples. Finally, the samples were dried at 50°C, for 1 h in an air-drying oven (Binder, Model FD 56, Germany). The dried samples were stored at −20°C until digestion *in vitro*.

### Digestion

*In vitro* digestion was carried out according to INFOGEST: static *in vitro* simulation gastrointestinal food digestion (19). In brief, prior to the experiment, the estimated activity of each enzyme used was analyzed: salivary amylase 1,600 U/mg, rabbit lipase 740 U/mg, pepsin 2,500 U/mg, and pancreatin 200 U/mg. Simulated saliva fluid (SSF), simulated gastric fluid (SGF), and simulated intestinal fluid (SIF) were freshly prepared by diluting the stock solution according to the experimental protocol.

All samples were prepared in triplicates. Two grams of the samples were weighted into a 50 mL tube. Three milliliters of preheated SSF were added and the samples were incubated for 2 min at 37°C. Six milliliters of SGF were added to the mixture. The pH of the samples was decreased to 3 with 6 N HCl. The gastric phase took 2 h at 37°C. Twelve milliters of SIF were added to the samples and the pH was increased to 7 with 1 M NaOH. The incubation took 2 h at 37°C. During digestion, the samples were continuously shaken (260 rpm) in a thermoincubator (Biosan, Latvia). Together with the samples, a blank (digestion electrolytes) and a blank with enzymes were prepared to determine the influence of these gastric buffers on each analysis. Samples were centrifuged and the supernatant in several aliquots was taken for further analysis. Digestion was terminated by shock-cooling. The samples were stored at −20°C until the analysis. All samples were analyzed within 1 week.

### Mineral Elements Analysis–AAS

Analysis of the Mg, Mn, Fe, Cu, and Zn content in extracts was determined using a 240 FS AA atomic absorption spectrometer (Agilent Technologies, Santa Clara, CA, USA) with flame atomization [acethylene–air flame (oxygen flow 13.5 L/min and acethylene 2.0 L/min)]. Standard solutions of Mg, Mn, Fe, Cu, Zn [1,000 mg/L (Merck, Germany)] were used to prepare the calibration solutions, which were acidified with 1% w:w concentrated suprapure HNO_3_. All solutions were prepared using demineralized water obtained with a Millipore Milli Q system (Millipore, Bedford, MA, USA). Samples and calibration solutions for the determination of Mg were diluted with 1% LaCl_3_ (Merck, Germany). The wavelength of the HCL lamp (Agilent Technologies, USA) for Mg was 285.2 nm, Mn was 279.5 nm, Fe was 271.90 nm, Cu was 324.7 nm, and for Zn was 213.9 nm. The concentration of mineral elements of digestion electrolytes (blank) was subtracted from sample data.

### Protein Analysis: Micro Lowry, Onishi, and Barr Modification

The assay was carried out according to Sigma Aldrich Total Protein Kit (product code TP0200 and B3934, Sigma Aldrich, USA). In brief, 400 μL of samples were diluted in 600 μL NaCl (0.85%). Then 200 μL of each sample or blank was transferred to a new tube. To each tube, 2.2 mL of Biuret reagent was added of, mixed well, and left to incubate for 10 min under laboratory temperature. Subsequently, 100 μL of Folin–Ciocalteau Phenol's reagent was added to the mixture and left to stand at the laboratory temperature for 60 min. Aliquots of 250 μL were transferred to the microtitrate plates (Nunc™ MicroWell™ 96-Well Microplates, Thermofisher Scientific, USA). The absorbance readings were carried out at 700 nm. Bovine albumin was diluted in 0.85% NaCl was used as a reference standard. As blank samples, 0.85% NaCl, the gastric buffer with enzymes, and the gastric buffer without enzymes were used.

### Amino Acid Analysis–GC-FID

Free amino acids were purified *via* SCX columns according to the manufacturer's protocol (Strata SCX, 55 μm, 70A, 500 mg/3 mL, Phenomenex, USA). Samples were derivatized according to the method by Husek and Sweeley (20). In brief, the amino acids residue was diluted with a mixture of water/ethanol/pyridine (60:32:8) and then, 5 μL of ethyl chloroformate was added to the mixture. The tube was shaken for 5 s and 100 μL of chloroform (containing 1% ethyl chloroformate) was added to each tube. An aliquot of chloroform layer was taken for the analysis. Separation of amino acids was conducted on a 30-m Zebron ZB-5HT Inferno capillary column of 0.25 mm i.d. and 0.25 μm film thickness (Phenomenex, USA) using nitrogen 5.0 as the carrier gas (Siad, Czech Republic). The injection volume was 1 μL and the flow rate was set as 3 mL/min. The injector temperature was 250°C with a split ratio of 20:1 and the FID temperature was 220°C. The oven temperature was programmed as follows: the column was held initially at 140°C for 1 min, then raised 40°C/min to 300°C. The temperature was held for 1 min. Chromatographic data were recorded and integrated using Clarity software (Data Apex, Czech Republic).

### Maltose, D-Glucose, Raffinose and Phytic Acid and Phosphorus Determination

For analysis, the Megazyme colorimetric assay kits for Raffinose/D-Galactose (K-RAFGA), Maltose/Sucrose/D-Glucose (K-MASUG), and Phytic Acid and Phosphorus (K-PHYT) were used (Megazyme, Bray, Ireland). Assays were carried out according to the manufacturer's protocols.

### Activity of Phytase

As a substrate, 7.5 mM sodium phytate in acetate 0.1 M buffer (pH 5.5) was used. The analysis was carried out in the microtitration plate. To each well was transferred 10 μL of the sample, and 170 μL substrate (0.5 mM sodium phytate in acetate 0.1 M buffer, pH 5.5) was added immediately or after 30 min. Samples were incubated at 37°C. The reaction was stopped by adding 10 μL of 1 M trichloroacetic acid to each well. Ninety microliters of the color reagent consisted of ascorbic acid (10 % w/v)/sulfuric acid (1 M) with ammonium molybdate (5 % w/v) at a final proportion of 1:5 in the mixture was added to each well. Simultaneously, samples without substrate and acetate buffer were prepared in the same way and used as a blank. H_3_PO_4_ in the acetate buffer was used as a phosphorus standard in the concentration range of 0–50 mM. The results were calculated according to equation (1) where the slope was calculated from phosphorus calibration equation and F is a dilution factor of the samples. Results were expressed as 1 U/mL, which is the amount of enzyme that releases 1 μM of inorganic phosphate from a sodium phytate substrate per minute at pH 5.5 and 37°C.


(1) $$\begin{array}{r} U/mL=\frac{\left(OD\;sample_{30\;min}-OD\;blank_{30\;min}\right)-\left(OD\;sample_{0\;min}-OD\;blank_{0\;min}\right)}{slope\;\times F} \end{array}$$

### Total Polyphenolic Compounds: Folin–Ciocalteau Reaction

The assay was modified into a microplate template; 10 μL of the sample was pipetted to 80 μL Folin–Ciocalteau reagent (10 times diluted by deionized H_2_O). After 5 min of incubation, 100 μL of (700 mM) Na_2_CO_3_ was added to each well. The absorbance at 700 nm was read after 15 min of incubation.

### Absorbance Measurements

Samples were placed in 96-well microtitration plates (Nunc™ MicroWell™ 96-Well Microplates, Thermofisher Scientific, USA). Measurements were performed at 22°C on the Synergy HTX microplate reader (Synergy HTX, Biotek USA).

### Data Treatment and Descriptive Statistics

The experimental work was carried out in three independent experiments. The obtained data is presented as an average value from three independent measurements. Results were analyzed using Shapiro–Wilk test, ANOVA, and Scheffe's Test. A significant result is considered at *p* < 0.05. The data was processed using Microsoft Excel® (USA).

## Results

### Protein Digestibility

**Figure 1:**
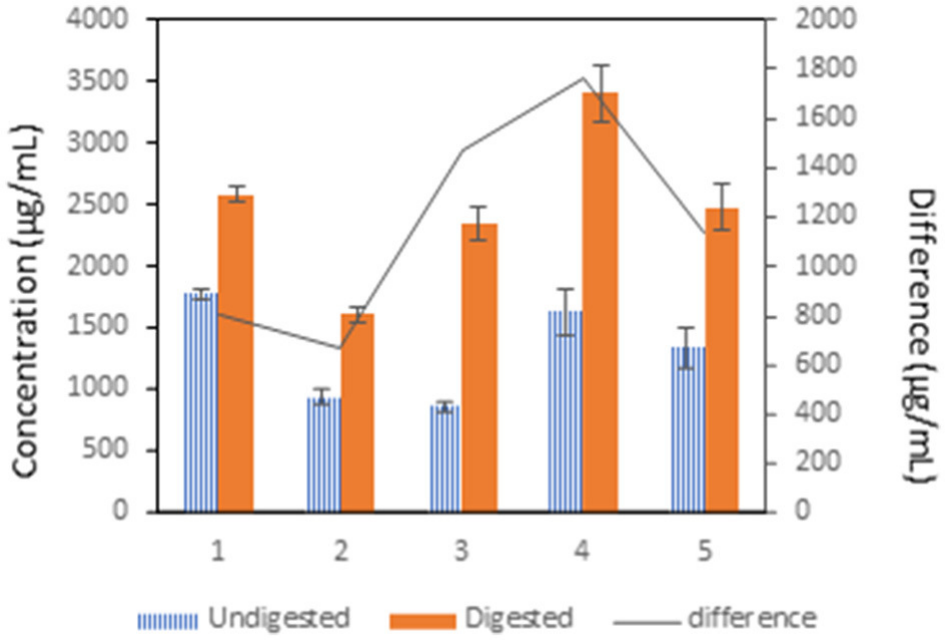
The influence of treatment of pea flour [(1): untreated flour, (2): cooked at 100°C for 10 min, (3): cooked at 100°C for 120 min, (4): fermented flour without cooking, (5): fermented flour cooked at 100°C for 120 min.] on protein digestion. The difference was calculated after subtraction of the concentration of the undigested sample from the digested sample.

Figure 1 shows a significant decrease in protein concentration of undigested variants in samples 2, 3, and 5 compared to the control group. After digestion, a significantly lower concentration was observed in sample 2 compared to the control sample. Sample 4 showed a significantly higher concentration of total proteins compared to the control sample. There was also a higher difference between undigested and digested protein concentration in samples 3, 4, and 5 compared to the control and sample 2.

The results obtained from gas chromatography of free amino acids analysis are presented in Table 2. Arrows mark a significant difference in each amino acid concentration from the control sample. The results showed a significant increase in the sums of all amino acids between undigested and digested variants for all samples. The concentration of free amino acids in their undigested and digested variants was significantly reduced in samples 2 and 3 compared to the control and samples 4 and 5.

**Table 2:** Released amino acids from pea flour before and after digestion.

**Sample**	**1**			**2**					**3**					**4**					**5**				
	**Undigested**	**Digested**	***diff***	**Undigested**		**Digested**		***diff***	**Undigested**		**Digested**		***diff***	**Undigested**		**Digested**		***diff***	**Undigested**		**Digested**		***diff***
**AA**	**μg/mg protein**		**%**	**μg/mg protein**				**%**	**μg/mg protein**				**%**	**μg/mg protein**				**%**	**μg/mg protein**				**%**
Phe	18 ± 2	37 ± 11	51	26 ± 11	↑	67 ± 24	↑	*61*	9 ± 2	↓	16 ± 4	↓	*44*	69 ± 17	↑	93 ± 14	↑	*26*	24 ± 3	↑	50 ± 10	↑	*52*
Ala	17 ± 4	26 ± 4	*35*	6 ± 0	↓	15 ± 1	↓	*60*	9 ± 1	↓	28 ± 5	–	*68*	20 ± 5	↓	54 ± 11	↑	*63*	23 ± 4	–	38 ± 7	↑	*39*
Gly	56 ± 3	175 ± 27	68	28 ± 7	↓	31 ± 4	↓	*10*	10 ± 1	↓	90 ± 23	↓	*89*	49 ± 8	–	147 ± 30	–	*67*	63 ± 11	–	114 ± 27	↓	*45*
Leu	914 ± 391	2724 ± 277	66	93 ± 8	↓	1792 ± 67	↓	*95*	35 ± 1	↓	4606 ± 273	↑	*99*	2225 ± 144	↑	14643 ± 2050	↑	*85*	3245 ± 559	↑	5686 ± 804	↑	*43*
Ile	475 ± 45	1405 ± 239	66	21 ± 3	↓	489 ± 58	↓	*96*	20 ± 5	↓	818 ± 104	↓	*98*	645 ± 122	↑	4630 ± 576	↑	*86*	1392 ± 198	↑	5460 ± 798	↑	*75*
Asn	7 ± 2	20 ± 2	*65*	10 ± 1	–	39 ± 7	↑	*74*	9 ± 2	–	25 ± 6	–	*64*	36 ± 3	↑	63 ± 17	↑	*43*	26 ± 3	↑	63 ± 13	↑	*59*
Asp	191 ± 32	629 ± 50	*70*	64 ± 18	↓	145 ± 19	↓	*56*	42 ± 11	↓	153 ± 14	↓	*73*	448 ± 85	↑	1401 ± 103	↑	*68*	262 ± 30	↑	1486 ± 332	↑	*82*
Met	390 ± 36	540 ± 69	*28*	24 ± 2	↓	200 ± 75	↓	*88*	19 ± 5	↓	52 ± 14	↓	*63*	583 ± 100	↑	1966 ± 524	↑	*70*	566 ± 79	↑	2828 ± 462	↑	*80*
Glu	241 ± 42	893 ± 108	*73*	91 ± 11	↓	302 ± 72	↓	*70*	60 ± 13	↓	270 ± 15	↓	*78*	691 ± 158	↑	7583 ± 702	↑	*91*	399 ± 68	↑	4746 ± 573	↑	*92*
Val	4746 ± 497	13631 ± 2185	*65*	918 ± 166	↓	9837 ± 548	↓	*91*	443 ± 66	↓	13662 ± 760	–	*97*	9777 ± 470	↑	19016 ± 1813	↑	*49*	7708 ± 531	↑	22494 ± 2267	↑	*66*
Cys	57 ± 6	99 ± 22	*42*	25 ± 3	↓	31 ± 7	↓	*19*	18 ± 4	↓	104 ± 20	–	*83*	837 ± 197	↑	1540 ± 322	↑	*46*	240 ± 35	↑	1314 ± 117	↑	*82*
Gln	4 ± 1	11 ± 2	*64*	2 ± 0	–	5 ± 1	↓	*60*	3 ± 1	–	7 ± 1	↓	*57*	42 ± 3	↑	108 ± 37	↑	*61*	18 ± 2	↑	66 ± 7	↑	*73*
Lys	91 ± 13	311 ± 39	*71*	99 ± 12	–	124 ± 14	↓	*20*	64 ± 9	↓	398 ± 88	↑	*84*	247 ± 18	↑	325 ± 66	–	*24*	204 ± 15	↑	282 ± 17	↓	*28*
His	331 ± 26	692 ± 172	*52*	111 ± 29	↓	351 ± 87	↓	*68*	119 ± 18	↓	250 ± 12	↓	*52*	286 ± 56	↓	364 ± 55	↓	*21*	201 ± 21	↓	322 ± 221	↓	*38*
Tyr	701 ± 75	1446 ± 139	*52*	181 ± 24	↓	853 ± 136	↓	*79*	162 ± 38	↓	1566 ± 379	–	*90*	317 ± 20	↓	1445 ± 206	–	*78*	225 ± 25	↓	845 ± 60	↓	*73*
Trp	1783 ± 892	5552 ± 600	*68*	1580 ± 183	–	2092 ± 155	↓	*24*	1514 ± 158	–	3513 ± 444	↓	*57*	4065 ± 435	↑	9844 ± 1853	↑	*59*	2844 ± 126	↑	10536 ± 535	↑	*73*
Sum of AA	10.02 ± 2.07	28.19 ± 3.95		3.28 ± 0.48		16.37 ± 1.28			2.54 ± 0.34		25.56 ± 2.16			20.34 ± 1.84		63.22 ± 8.38			17.44 ± 1.71		56.33 ± 6.25		
(mg/mg																							
protein)																							

### Starch Digestibility

**Figure 2:**
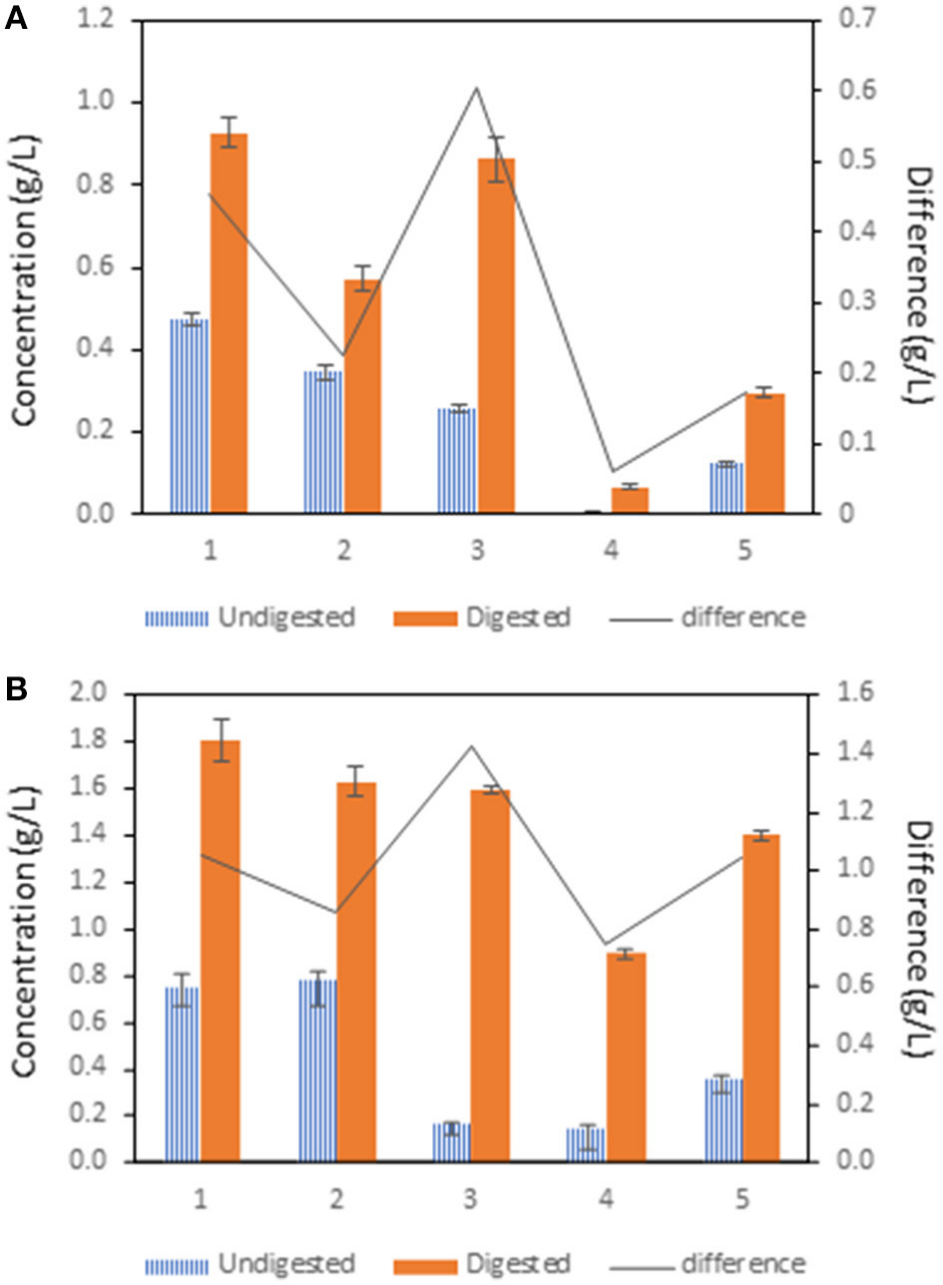
The influence of treatment of pea flour [(1): untreated flour, (2): cooked at 100°C for 10 min, (3): cooked at 100°C for 120 min, (4): fermented flour without cooking, (5): fermented flour cooked at 100°C for 120 min.] on saccharide digestion. The difference was calculated after subtraction of the concentration of the undigested sample from the digested sample. **(A)** glucose, **(B)** maltose.

Figure 2A presents changes in maltose concentration before and after digestion. A significant decrease in maltose concentration in the undigested samples 3, 4, and 5 was evident. A significantly lower maltose concentration was found in sample 4 and significantly higher concentration in digested variants of maltose was found in sample 5, both compared to the control sample. Sample 3 showed a significant difference in maltose concentration between the undigested and digested variant.

From the graph in Figure 2B, it is evident that there was a significant decrease in glucose concentration in samples 2–5 compared to the control in undigested variants. Samples 4 and 5 showed a significantly reduced glucose concentration compared to the undigested samples 2 and 3. After digestion, there was a significant decrease in glucose concentration in samples 2, 4, and 5 compared to the control sample. The difference between undigested and digested samples was significantly higher in sample 3. In samples 2, 4, and 5 the difference was significantly lower compared to the control.

### Antinutritional Compounds

**Figure 3:**
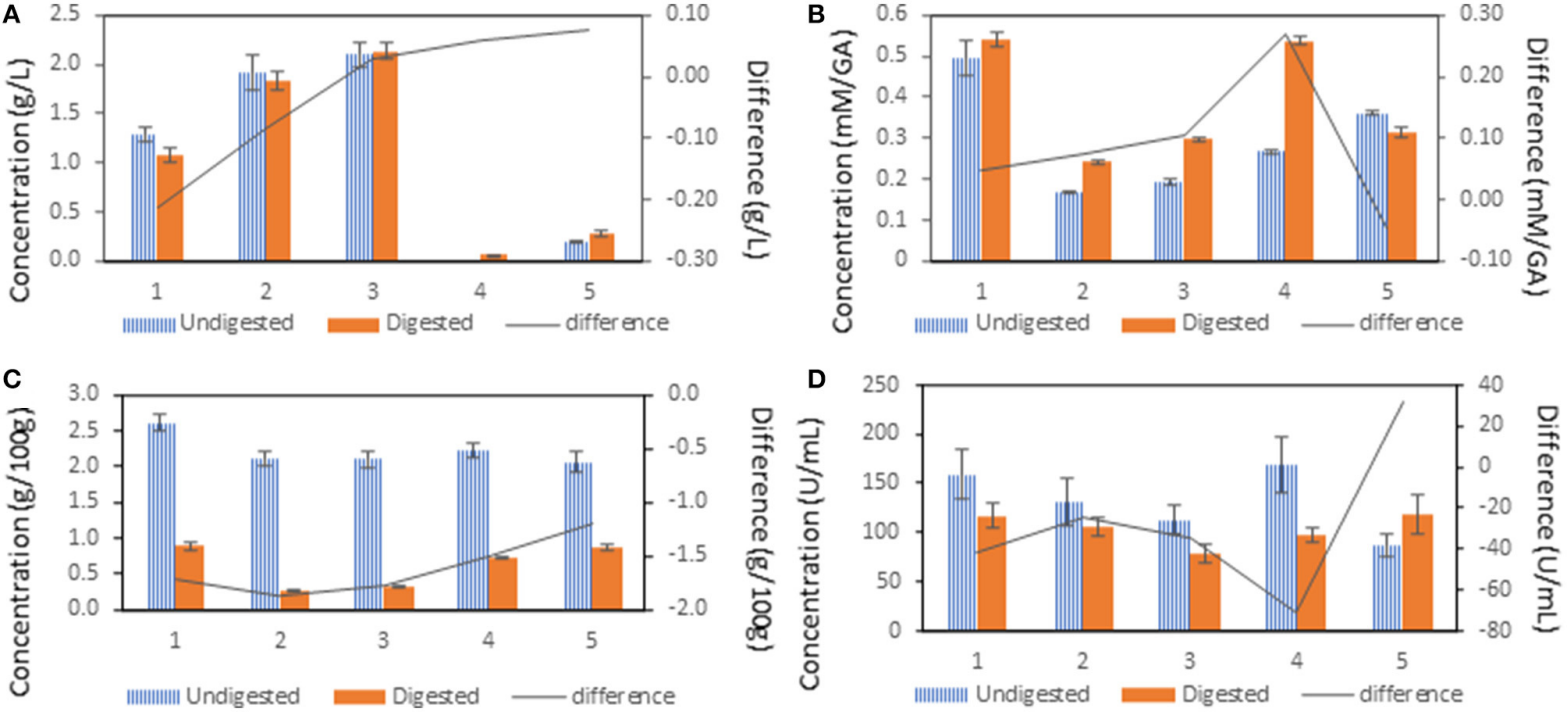
The influence of treatment of pea flour [(1): untreated flour, (2): cooked at 100°C for 10 min, (3): cooked at 100°C for 120 min, (4): fermented flour without cooking, (5): fermented flour cooked at 100°C for 120 min.] on antinutritional compounds. The difference was calculated after subtraction of the concentration of the undigested sample from the digested sample. **(A)** raffinose, **(B)** polyphenols, **(C)** phytic acid, **(D)** phytase.

From the graph in Figure 3A, it can be seen that the raffinose content did not change during the digestion, except in sample 4. A higher concentration in digested variant was determined because the raffinose concentration in the undigested sample 4 was below the detection limit. The difference between undigested and digested variants was not significant in other samples. The concentration of raffinose in both undigested and digested variants was significantly higher in sample 2 and in sample 3 compared to the control sample. The fermented samples 4 and 5 showed a significantly lower raffinose concentration in both undigested and digested variants compared to the control samples.

From the results shown in Figure 3B, it is evident that the control sample had a significantly higher polyphenol concentration compared to samples 2–5 in both undigested and digested variants except for the digested variant of sample 4. The level of polyphenols in undigested variants was significantly lower in samples 2 and 3 compared to samples 4 and 5. Sample 5 showed a significantly higher polyphenols level compared to sample 4. The digested variants of samples 2–4 showed a significantly higher concentration of total polyphenols compared to their undigested variants, whereas sample 5 showed the opposite trend.

The data in Figure 3C shows that the concentration of phytic acid in undigested variants was significantly lower in all treated samples (2–5) compared to the control sample. After digestion, the concentration of phytic acid was significantly lower in Samples 2 and 3 compared to the control sample and Samples 4 and 5. The difference in phytic acid concentration between undigested and digested variants was highest in the control sample.

As shown in Figure 3D, phytase activity showed no significant changes between the digested variants in all samples. A significant decrease in the phytase activity in undigested variant was observed only in sample 5 compared to the control sample. The difference in phytase activity between the control sample and samples 2, 3, and 4 was not observed. The difference between phytase activity in the undigested and digested variant of each sample was significantly higher in sample 4.

### The Concentration of Free Mineral Elements

**Figure 4:**
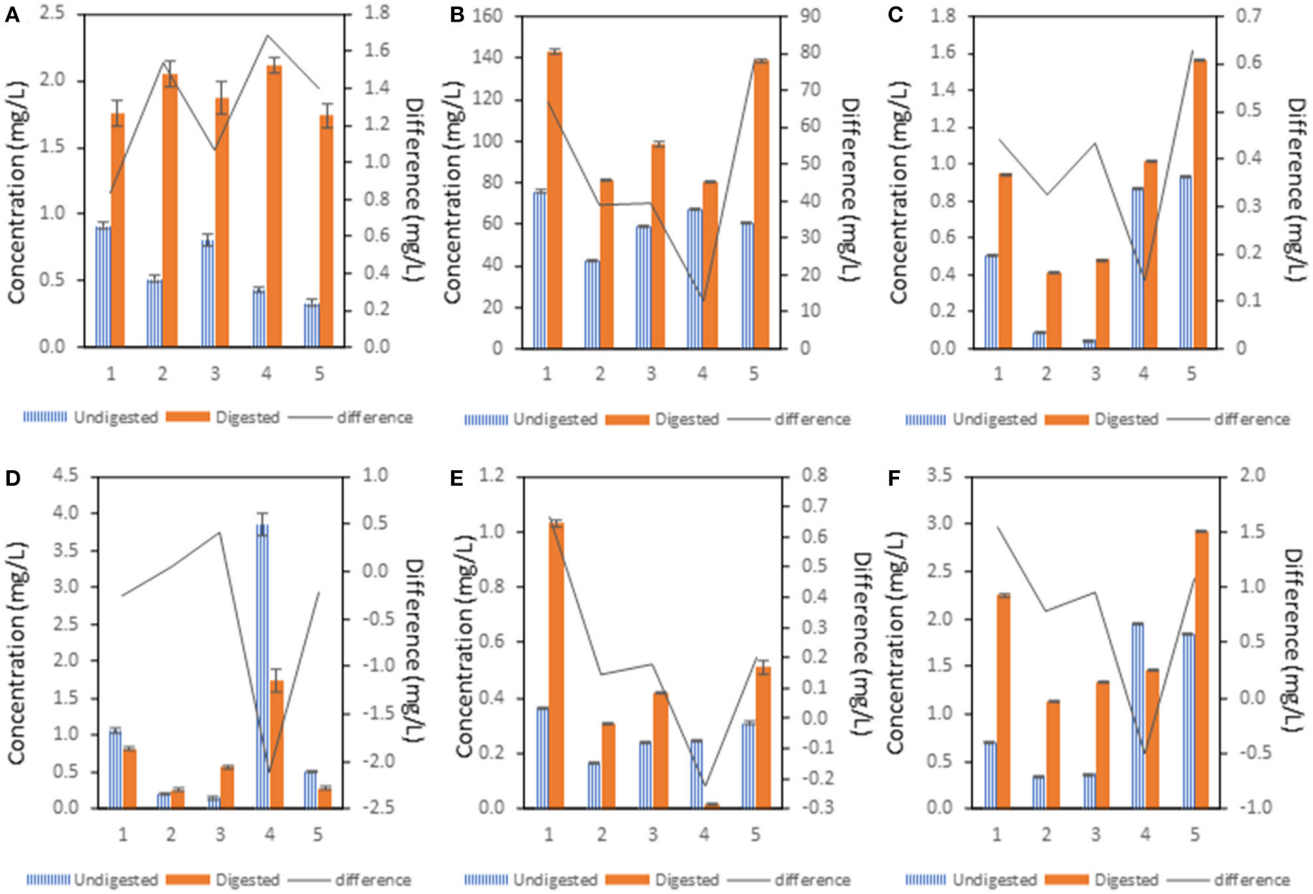
The influence of treatment of pea flour [(1): untreated flour, (2): cooked at 100°C for 10 min, (3): cooked at 100°C for 120 min, (4): fermented flour without cooking, (5): fermented flour cooked at 100°C for 120 min.] on mineral concentration. The difference was calculated after subtraction of the concentration of the undigested sample from the digested sample. **(A)** P, **(B)** Mg, **(C)** Mn, **(D)** Fe, **(E)** Cu, **(F)** Zn.

From the graph in Figure 4A, it is obvious that P concentration in all the samples was significantly higher in the digested variants compared to the undigested variants. The significantly lower P concentration in undigested variants compared to the control sample was determined in samples 4 and 5. Digested samples showed no significant change in the P concentration compared to control.

From Figure 4B, it can be seen that Mg concentration was significantly higher in all digested variants compared to the undigested variants. The greatest difference in Mg concentration between undigested and digested variants was observed in the control sample and sample 5. The smallest difference was found in sample 4. Samples 2–5 showed significantly lower Mg concentration compared to control in undigested variants. Samples 2–4 showed significantly lower concentration of Mg compared to control in digested variants.

Figure 4C shows that undigested variants had a significantly lower Mn concentration in all samples compared to the digested variants. The largest difference was observed in sample 5 in contrast to sample 4 which showed the smallest difference. Samples 2 and 3 showed a significantly lower Mn concentration in digested and undigested variants compared to the control. Samples 4 and 5 showed a significantly higher Mn concentration in undigested variants compared to the control sample. In addition, sample 5 showed a significantly higher Mn concentration compared to the control in the digested variant.

From Figure 4D, it is obvious that Fe concentration in undigested samples showed a significant decrease in samples 2, 3. and 5 compared to the control. Undigested and digested sample 4 showed a significantly higher Fe concentration compared to the control sample and other samples. Unlike sample 2, there was a significant difference between undigested and digested variants in the Fe concentration. The highest difference between Fe concentration of undigested and digested variant was shown in sample 4.

Figure 4E shows a significant decrease in Cu concentration in samples 2–5 compared to the control in the undigested variant as well as in the digested variants. A significant difference between the undigested and the digested variant was found in all samples and the control sample.

As shown in Figure 4F, the undigested variants of samples 2 and 3 showed a significantly lower concentration of Zn compared to the control. On the contrary, samples 4 and 5 in undigested variants showed a significantly higher concentration of Zn compared to the control sample. Samples 2–4 showed a significantly lower Zn concentration in the digested variant compared to the control. Only in sample 5 there was a significantly higher Zn concentration in the digested variant compared to the control and other samples. There were significant differences in Zn concentration between the undigested and digested variants in all samples including the control.

## Discussion

The digestion of food depends on several internal and external factors that influence the bioavailability and bioaccessibility of nutrients. Although, the digestion process is complex and hardly reproductible *in vitro*, this practice can be useful in the terms of the evaluation of suitable food preparation, especially when considering antinutritional factors (19).

### Protein Digestibility

The results clearly demonstrate increased protein concentration in digested samples, which is caused by protein breakdown and accessibility of the peptides to the reagent. The lowest protein concentration was observed in samples 2 and 3 after cooking. Generally, it is known that food processing triggers the breakdown of molecular structures and allows the digestive enzymes to access nutrients. During cooking, the proteins are denatured and unfolded, which leads to their better digestibility (21). However, the protein denaturation opens the hydrophobic protein clusters, which can aggregate and decrease digestibility (22). Also, the formation of disulphide bridges between amino acids leads to the decrease of protein digestibility as well as racemization and formation of Maillard reaction products (23). From the results of this study, it is clear that the denatured proteins in treated samples before digestion were less accessible to pepsin and pancreatin. The obtained findings corroborate the findings of Sousa et al., who determined the resistance of vicilin, provicilin, convcilin, legumin A, and legumin A2 to gastric pepsin *in vitro*, which are the major proteins in the peas (24). These proteins belong to the group of globulins (8). It has been shown the globulins and also β-sheet structured proteins and hydrophobic proteins are not completely digested and cleave into amino acids (25). On the contrary, the results do not match the findings of Byanju et al. who observed higher protein digestibility in green peas after cooking. But, as the authors state, this could have been caused by sterilization of the pea flour before treatments, which could break down the pea flour matrix structure (26). Not only heat treatment could influence the protein digestibility, but also, the presence of antinutritional compounds such as tannins, lectins, saponins, phytate, trypsin, and chymotrypsin inhibitors which are present in pea four could prevent protein hydrolysis (27). A strong dependence was found between protein and polyphenols concentration in undigested variants of samples 1–3 (*R*^2^ = 0.9806); however, in the digested variants, the dependence was not strong (R^2^ = 0.6492). In the terms of fermented samples (4 and 5), the protein concentration was higher in comparison with the control sample. A possible explanation of this result might be an increase of microbial mass and thus an increase of total protein. The possible explanation could be that *L. plantarum* might have disrupted polysaccharide matrix from pea flour and enabled digestive enzymes to access storage proteins. This phenomenon could also explain the increased protein concentration in digested samples. The concept of the usage of bacteria to increase the biomass in the starchy food is not new. Coghetto at al. used *L. plantarum* BL011 in a new, completely animal-free medium in bioreactors with a soybean medium. A maximal yield of biomass of 17.87 g/L was observed, whereas lactic acid, the most important lactic acid bacteria metabolite, peaked at 37.59 g/L, corresponding to a productivity of 1.46 g/L/h (28). Studies on the production of a microbial protein state that bacterial biomass total protein content ranges from 50 to 83 % (29).

To confirm the digestion of proteins, amino acid analysis was carried out by gas chromatography. This method is sensitive and able to detect free amino acids at trace levels (20); however, it has limitations in the determination of arginine, threonine and it is less sensitive to serine. In undigested pea flour (samples 1–3) there were found the highest concentration of Val, Leu, Trp, Tyr, respectively. In fermented samples there were further increased concentrations of Cys, Met, and Glu compared to non-fermented samples. As a sum of all amino acids, there is an evident increment of free amino acids in the undigested and digested samples compared to the control and samples 2 and 3. The highest difference between the digested and undigested samples of free amino acids was observed in sample 3. The findings showed the release of amino acids was dependent on the pea flour preparation and fermentation. The results are consistent with the study of Thompson et al. who found that the fermentation of beans and cauliflower increased the concentration of alanine, glycine, histidine, isoleucine, leucine, and valine (30). However, in contrast to this study, it was additionally observed that there was an increase of Trp, Lys, Cys, and Met and a decrease of His. It has been shown that the fermentation processes cause changes in the pea flour matrix, which allows or blocks digestive enzymes' access to the proteins. The differences in the free amino acid release are also caused by various abilities of digestive enzymes to cleave proteins on their specific sites during the digestion process (24). It is generally accepted that pepsin prefers to cleave hydrophobic amino acid residues and it is specific for phenylalanine, leucine, tryptophan, and tyrosine in position P1, whereas histidine and lysine cleave rarely. Trypsin preferentially cleaves at arginine and lysine (31). There are several cleavage rules which could be found e.g., in expasy.org (32).

### Starch Digestibility

Starch is the main stock polysaccharide in peas and ranges between 50 and 60% depending upon the variety (33). Structurally, legume starch has a higher amylose content (30–40% of amylose and 60–70% of amylopectin) compared to other starchy plants. It has been shown that amylose is less easily digestible than amylopectin, probably due to the much larger surface area of the molecule (34). In the experiment, the maltose and glucose assays were carried out to evaluate the rate of the peas' starch digestion. The results showed an increase of glucose and maltose in all digested variants. The highest difference between digested and undigested variant was seen in sample 3. One possible explanation is the formation of retrograded starch in undigested samples, which was less accessible in the maltose and glucose assay. Blazek et al. observed that during food processing, starch granules swell and lose their crystallinity and molecular organization in a process called gelatinization. Irreversible changes are linked to the disruption of molecular structure of amylose and amylopectin. When degraded starch is exposed to the digestive enzymes, the rate of starch cleavage by α-amylase is much higher compared to the native starch (35). But after the cooling, drying, and storage of the gelatinized starch, amylose and amylopectin reorder their structure, which is not recognized by digestive enzymes and thus becomes less digestible (36). Surprisingly, the significant increase of the maltose content in sample 3 compared to other samples (Figure 2B) was observed only after digestion. This could be due to pH changes during the *in vitro* digestion as well as improving the bioaccessibility of starch from the activity of other digestive enzymes. The comparison of the control sample and cooked samples with their fermented variants showed maltose and glucose content is significantly lower in undigested and digested samples. These findings support a study by Florencio et al. who demonstrated the presence of *L. plantarum* amylolytic enzymes and the ability of *L. plantarum* to cleavage starch and metabolize glucose (37). Moreover, it has been observed that the maltogenic amylase from *L. plantarum* is able to hydrolyze the long side chains of amylopectin, which contributed to slowing the retrogradation of starch in a bread (16). The estimated maximum total amylase and α-amylase production of *L. plantarum* CGMCC 14177 were 286.8 and 208.1 U/g, respectively (38). Taken together, these results indicate that *L. plantarum* decreases the content of digestible starch and simultaneously increases digestible proteins.

### Antinutritional Compounds

In addition, the *L. plantarum* showed the ability to metabolize raffinose. Since the human digestive system does not have an enzyme for raffinose cleavage, its digestion takes a place *via* intestinal microbiota (39). Thus, the digestion of the oligosaccharides which belong to FODMAPs, peas, and other legumes can have bloating effects. As the results show, the difference provided between undigested and digested variants was not clearly observed, but there is strong evidence of the ability of *L. plantarum* to utilize raffinose in fermented samples. Moreover, a significant difference in raffinose concentration was observed between uncooked sample 4 and cooked sample 5 (Figure 3A). A possible explanation for these results may be that residual raffinose was released from the pea flour by cooking. These results are consistent with those of other studies and suggest that *L. plantarum* significantly decreased the content of raffinose and other FODMAPs in starchy foods (40–42).

Peas are rich in phytochemicals, mostly phenolic, terpenoids, and nitrogenous compounds which have shown antioxidant activities (43). Their higher content is in the hulls, which are usually separated during the processing of peas. In terms of pea flour digestion, the tannins impair protein digestion, forming a protein precipitate and decreasing protein bioaccessibility. For this reason, it is advisable to cook peas to reduce the content of tannins (44). Based on the obtained results, the total polyphenols significantly decreased in cooked samples 2 and 3, as well as in sample 5 when compared to sample 4. Surprisingly, the content of polyphenols was higher in the fermented samples, which supports findings of other studies where researchers elucidated the production of polyphenolic and antioxidant substances as secondary metabolites from *L. plantarum* (45, 46). In our experiment, a strong dependence (R^2^ = 0.9087) of polyphenols and protein concentration in digested variants of samples 2–5 was observed, which supports the idea of diminishing protein digestion in the presence of polyphenols.

The primary widely-recognized antinutrient compound in peas is phytic acid (47). A large body of studies have reported that phytate acts as an inhibitor to the absorption of mineral elements such as Zn, Fe, and Ca, as well-acting to decrease protein availability (48). In part, the phytate is cleaved by endogenous phytase, but for nutritional purposes, there are several approaches to reduce phytate, including soaking, germination, milling, or fermentation. While phytate is thermo-stabile, several studies have confirmed no effect on phytate reduction during traditional cooking (49). The content of phytic acid was significantly decreased in all treated and undigested samples, and there was a significant decrease in phytic acid after digestion. This could be contributed to the activity of native phytase when the activity of phytase and the concentration of phytic acid is in mutual dependence in samples 2–5 (*R*^2^ = 0.9539) in their undigested variants. On the other hand, the activity of phytase did not show significant changes among treated samples both in undigested and digested variants (Figure 3D). In contrast to the results obtained from this study, fermentation had no effect on the content of phytic acid. It has been shown *L. plantarum* fermentation significantly decreased (~35%) the phytic acid content in barley, coprecipitate, green gram, and tomatoes food mixture (50). Byanju et al. also have not proved the reduction of phytic acid by the fermentation of green peas, but rather in modified soybeans and lentils (26). *L. plantarum* has phytase activity, although it is very low to utilize the content of phytic acid completely (51). Overall, fermentation conditions as well as substrate may play a key role in phytic acid degradation by microbial fermentation. In future work, it would be appropriate to focus on presoaking peas or to coferment pea flour with phytase-producing microorganisms (52).

### The Concentration of Free Mineral Elements

To evaluate the bioaccessibility of mineral compounds, the concentration of each mineral in the supernatant was determined. The results suggest that the treatment of the pea flour bears significant influence on the bioaccessibility of minerals. Cooking (samples 2 and 3) caused a significant decrease of proposed minerals compared to the control sample, and this effect is noticeable in both digested and undigested variants. Several studies have confirmed cooking of pulses significantly decreases the bioaccessibility of minerals due to their interaction with antinutritional compounds such as phytate and pectin or fiber (23, 48). One suggested mechanism for this effect is the solubilization of mineral chelators which become more accessible for minerals during the cooking time (53, 54). Noteworthy changes were observed in the fermented pea flour (samples 4 and 5) in terms of mineral compounds release. In the case of P, a significant decrease in the content of P in both samples 4 and 5 in their undigested variants was found. In sample 5, there was a significant decrease in Fe concentration, whereas in sample 4 the Fe concentration was strongly increased as well as Mn, Cu and Zn, again compared to the control sample in undigested variants. The same trend was found for their digested variants, except for Cu in sample 4 (Figure 4). Most of the examined minerals were released after the pea flour digestion, except sample 4 in the case of Fe, Cu, and Zn and sample 5 in the case of Fe. Moreover, there was observed an adverse proportion of the content of these minerals released after digestion. The increased concentration of free minerals available to the GIT after digestion can be explained by the activity of digestive enzymes that released them from pea flour, but detailed studies are lacking to confirm the obtained results from this experiment. Similar trends were shown in the study of Sindhu et al. They observed the improved HCl-extractability of iron (54–67%), calcium (22–32%), sodium (25–30%), and potassium (17–24%) after probiotic fermentation of a naturally developed food blend (54). Unfortunately, there is a lack of literature to clarify our results, indicating that further research on this topic will be needed. Several randomized controlled trials (55–57) have shown that *L. plantarum* strongly increased the intestinal iron uptake from a diet with a low dose of iron (58). The mechanism has not been fully understood yet, but some studies indicate several mechanisms at the level of the microorganism itself, in conjunction with interactions in the GIT that are likely to act synergistically: (1) *L. plantarum* increases levels of a ferric reductase in human intestinal cells, enhances mucin production and suppresses hepcidin, the regulator of systemic iron homeostasis; (2) production of p-hydroxyphenyllactic acid can promote the reduction of ferric iron to the more bioavailable ferrous form (59). Surprisingly, sample 5 showed a significantly lower Fe concentration in both variants, which could mean that the mechanism of increasing the concentration of free Fe is dependent on temperature. Although this study was carried out *in vitro*, the results suggest that there may be another mechanism that *L. plantarum* uses to increase the bioaccessibility of iron as well as other minerals.

## Conclusion

The aim of the present research was to provide a comprehensive view of protein and starch digestibility, and to determine the content of antinutritional compound as well as mineral elements in cooked and fermented pea flour by *L. plantarum*. This study showed that protein and starch digestibility was significantly reduced after 10 min of cooking, whereas significantly improved after 120 min of cooking and the lactic fermentation. In addition, the lactic fermentation improved the nutritional quality of pea flour. The higher content of Cys, Met and Gln and the bioaccessibility of Fe, Mn, Mg, and Zn was observed when compared to non-fermented samples. Also, raffinose content significantly decreased. Taken together, lactic fermentation offers an extensive way to create functional foods from peas.

## Data Availability Statement

The raw data supporting the conclusions of this article will be made available by the authors, without undue reservation.

## Author Contributions

SS: conceptualization, methodology, validation, investigation, data curation, writing–original draft, and visualization. AR: investigation, data curation, and writing–review and editing. PSl: writing–review and editing and validation. JS: writing–review and editing, formal analysis, data curation, and validation. PSk, JH, and RD: writing–review and editing. IS: writing–review and editing, project administration, and funding acquisition. PH: writing–review and editing and supervision. All authors contributed to the article and approved the submitted version.

## Funding

This study was funded by the project development of Biofortified Pea Breeding Lines with Low Phytic Acid Content, Project Number: QK1810072.

## Conflict of Interest

IS, JH, and RD were employed by Agritec Plant Research, Ltd. The remaining authors declare that the research was conducted in the absence of any commercial or financial relationships that could be construed as a potential conflict of interest.

## Publisher's Note

All claims expressed in this article are solely those of the authors and do not necessarily represent those of their affiliated organizations, or those of the publisher, the editors and the reviewers. Any product that may be evaluated in this article, or claim that may be made by its manufacturer, is not guaranteed or endorsed by the publisher.
